# Supplementary material for: Genomic basis underlying the metabolome-mediated drought adaptation of maize
Source: Genome Biol. 2021 Sep 6;22:260. doi: 10.1186/s13059-021-02481-1 (PMC8420056; doi:10.1186/s13059-021-02481-1)
Supplement: Supplementary file 1 — Additional file 1: Figures S1-S14. [file 13059_2021_2481_MOESM1_ESM.pptx]

## Slide 1
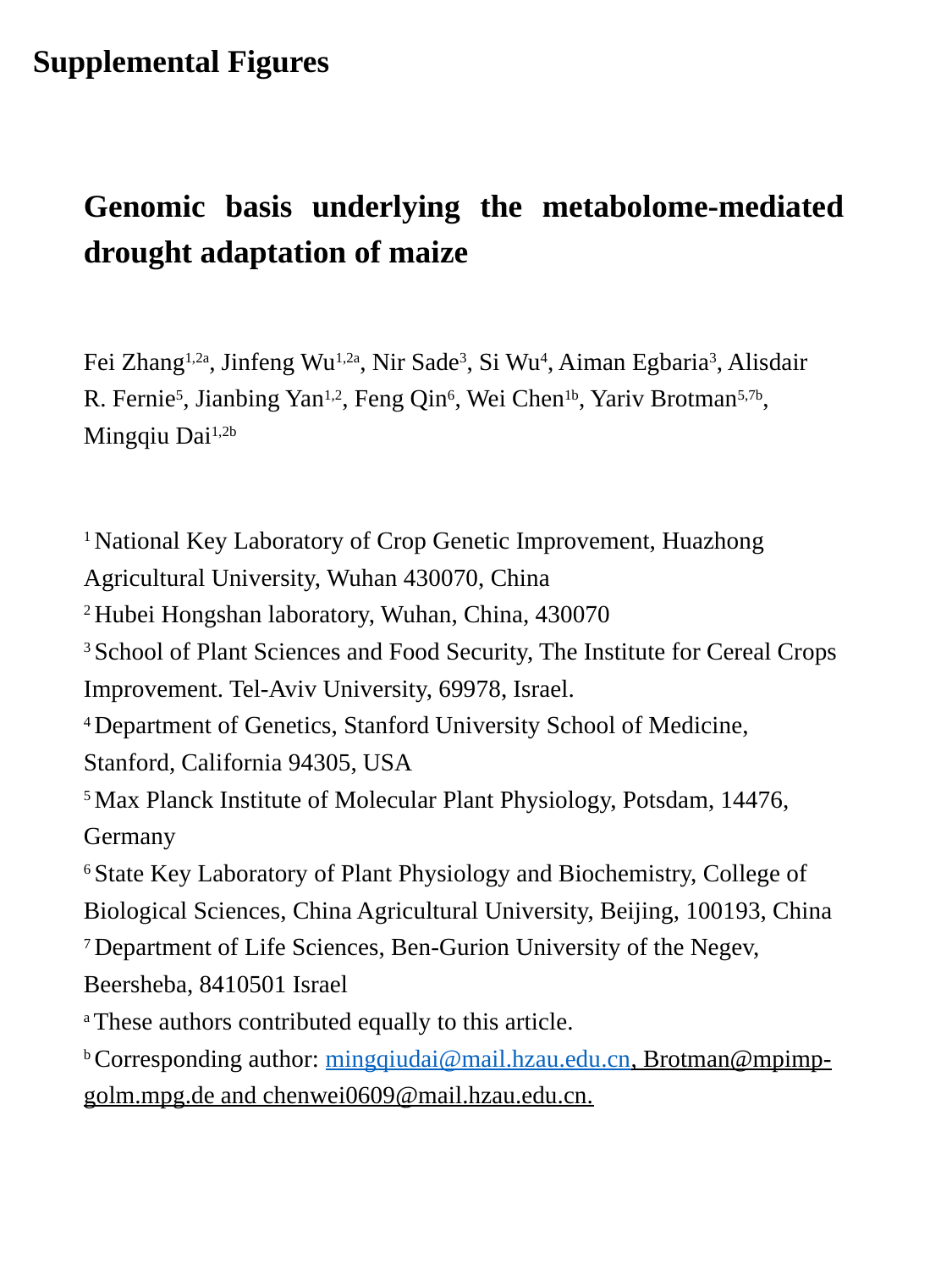

Supplemental Figures
Genomic basis underlying the metabolome-mediated drought adaptation of maize
Fei Zhang1,2a, Jinfeng Wu1,2a, Nir Sade3, Si Wu4, Aiman Egbaria3, Alisdair R. Fernie5, Jianbing Yan1,2, Feng Qin6, Wei Chen1b, Yariv Brotman5,7b, Mingqiu Dai1,2b
1 National Key Laboratory of Crop Genetic Improvement, Huazhong Agricultural University, Wuhan 430070, China
2 Hubei Hongshan laboratory, Wuhan, China, 430070
3 School of Plant Sciences and Food Security, The Institute for Cereal Crops Improvement. Tel-Aviv University, 69978, Israel.
4 Department of Genetics, Stanford University School of Medicine, Stanford, California 94305, USA
5 Max Planck Institute of Molecular Plant Physiology, Potsdam, 14476, Germany
6 State Key Laboratory of Plant Physiology and Biochemistry, College of Biological Sciences, China Agricultural University, Beijing, 100193, China
7 Department of Life Sciences, Ben-Gurion University of the Negev, Beersheba, 8410501 Israel
a These authors contributed equally to this article.
b Corresponding author: mingqiudai@mail.hzau.edu.cn, Brotman@mpimp-golm.mpg.de and chenwei0609@mail.hzau.edu.cn.

## Slide 2
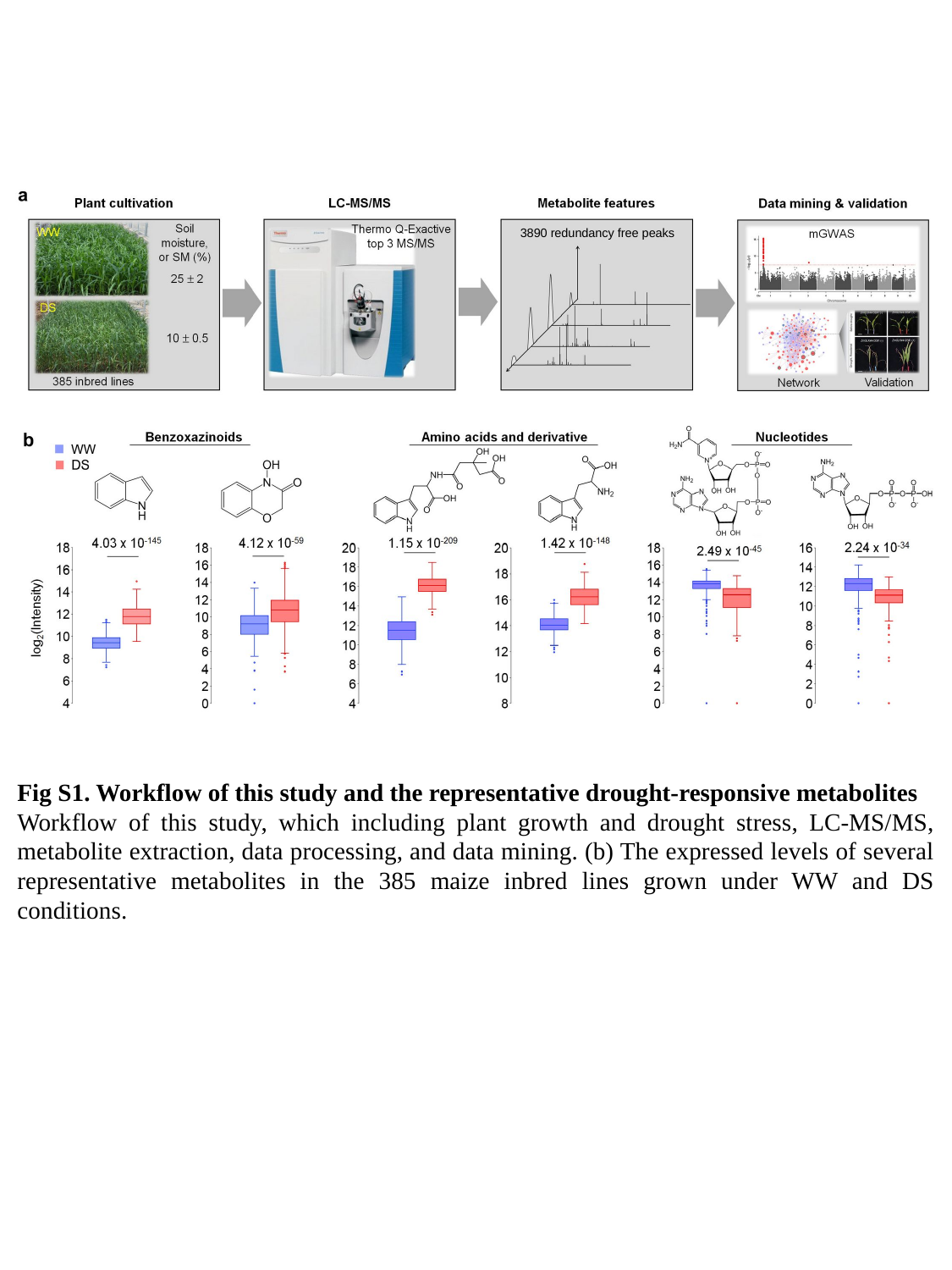

3890 redundancy free peaks
Fig S1. Workflow of this study and the representative drought-responsive metabolites
Workflow of this study, which including plant growth and drought stress, LC-MS/MS, metabolite extraction, data processing, and data mining. (b) The expressed levels of several representative metabolites in the 385 maize inbred lines grown under WW and DS conditions.

## Slide 3
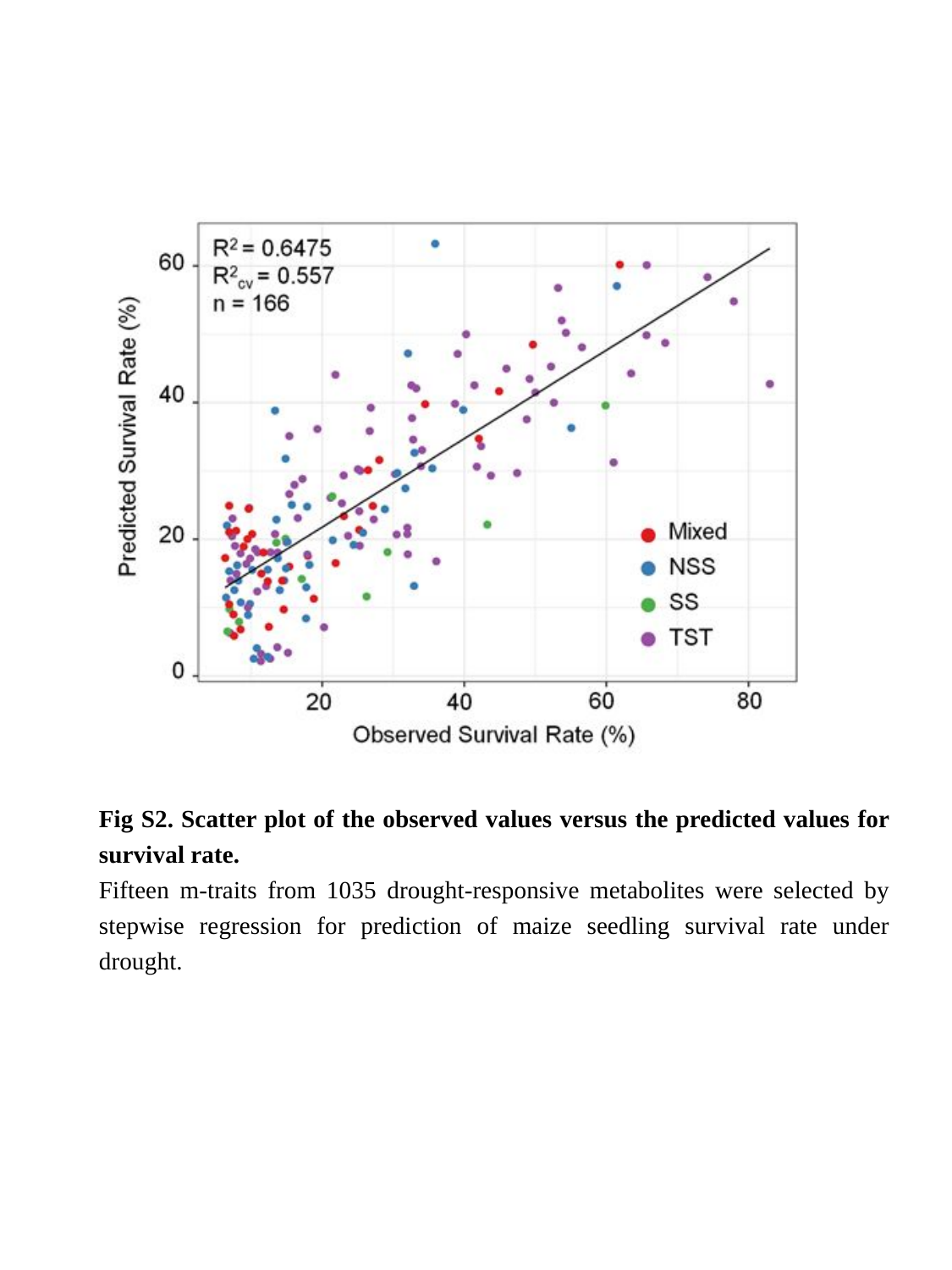

Fig S2. Scatter plot of the observed values versus the predicted values for survival rate.
Fifteen m-traits from 1035 drought-responsive metabolites were selected by stepwise regression for prediction of maize seedling survival rate under drought.

## Slide 4
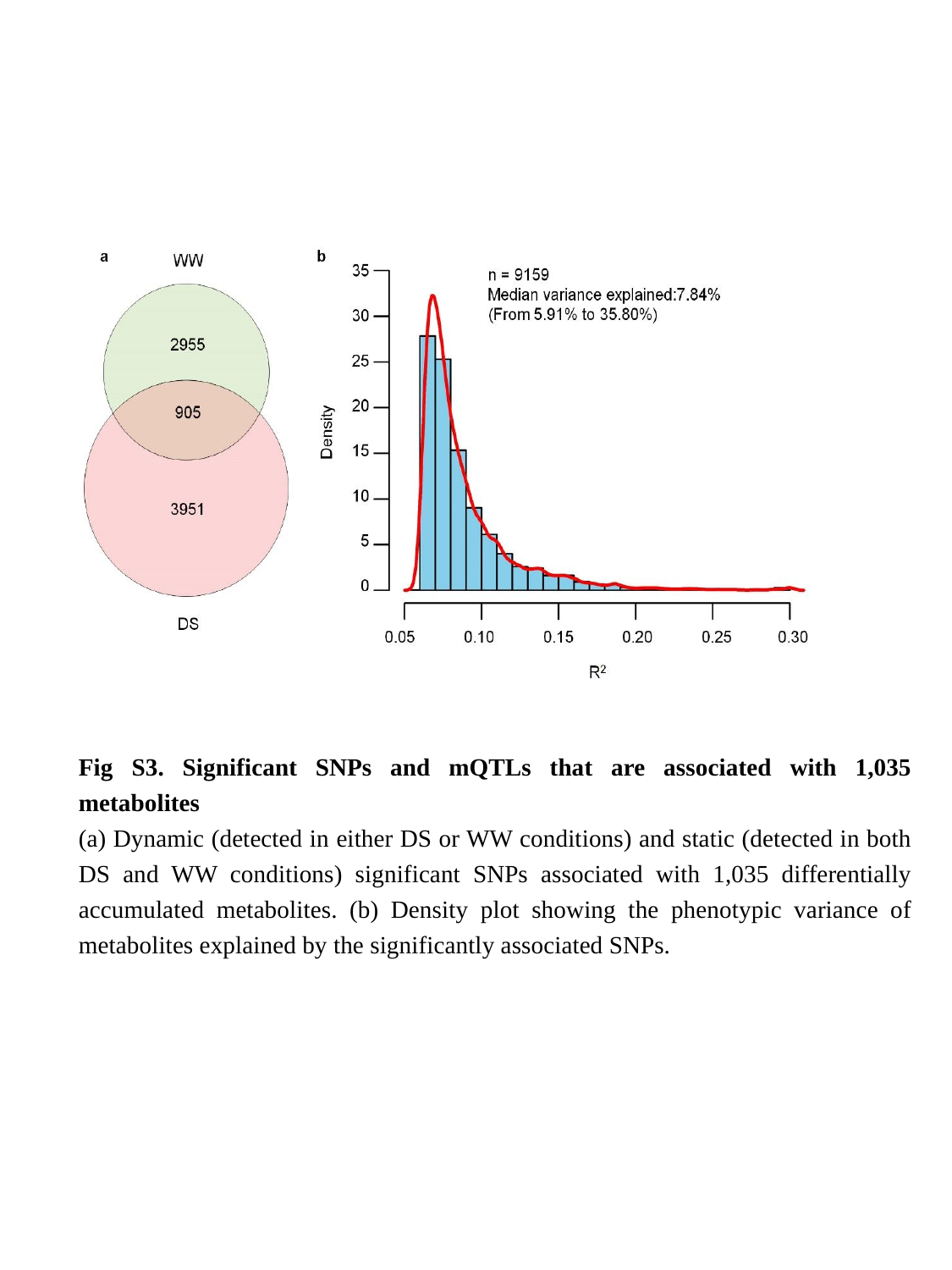

Fig S3. Significant SNPs and mQTLs that are associated with 1,035 metabolites
(a) Dynamic (detected in either DS or WW conditions) and static (detected in both DS and WW conditions) significant SNPs associated with 1,035 differentially accumulated metabolites. (b) Density plot showing the phenotypic variance of metabolites explained by the significantly associated SNPs.

## Slide 5
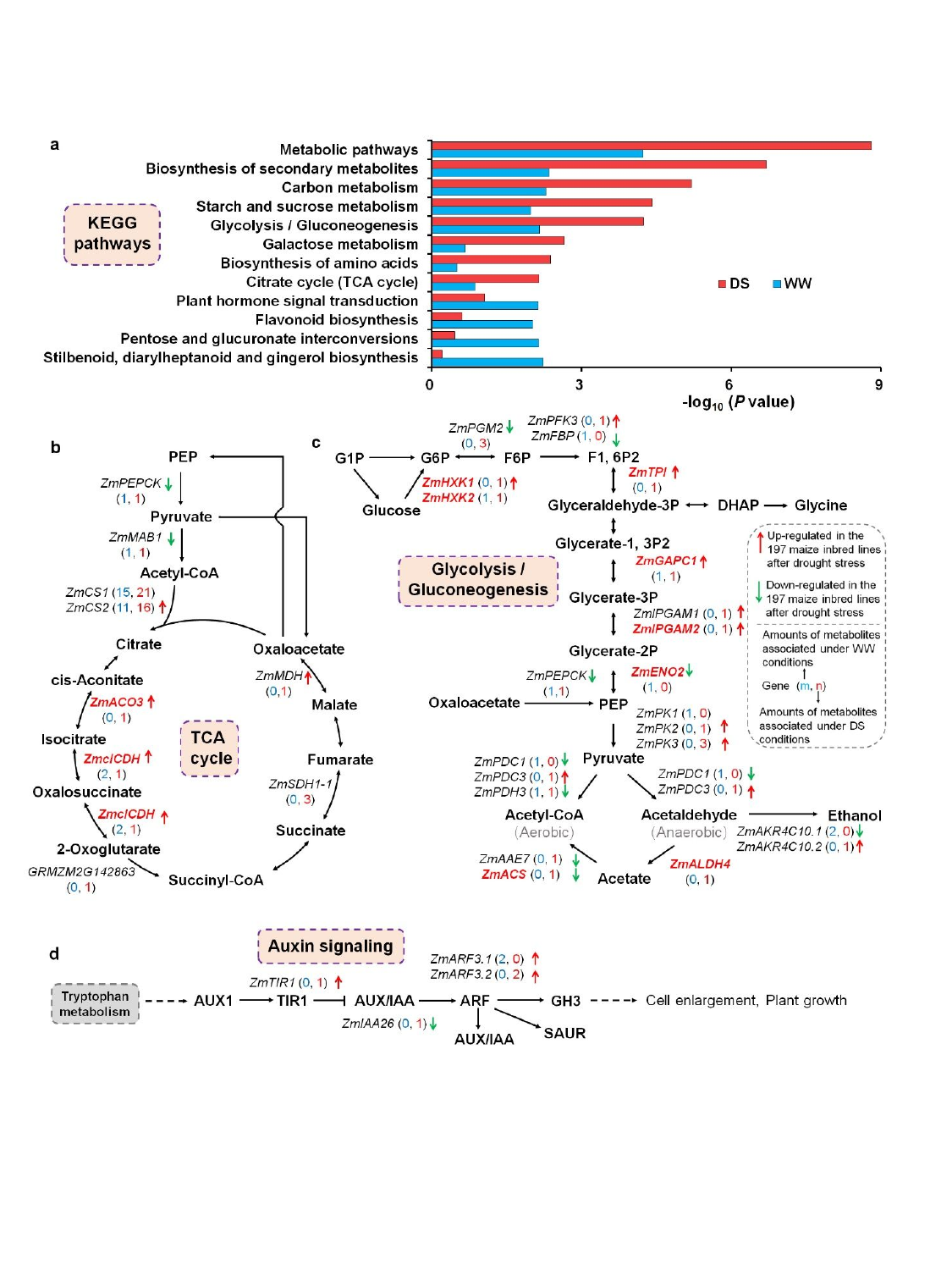

## Slide 6
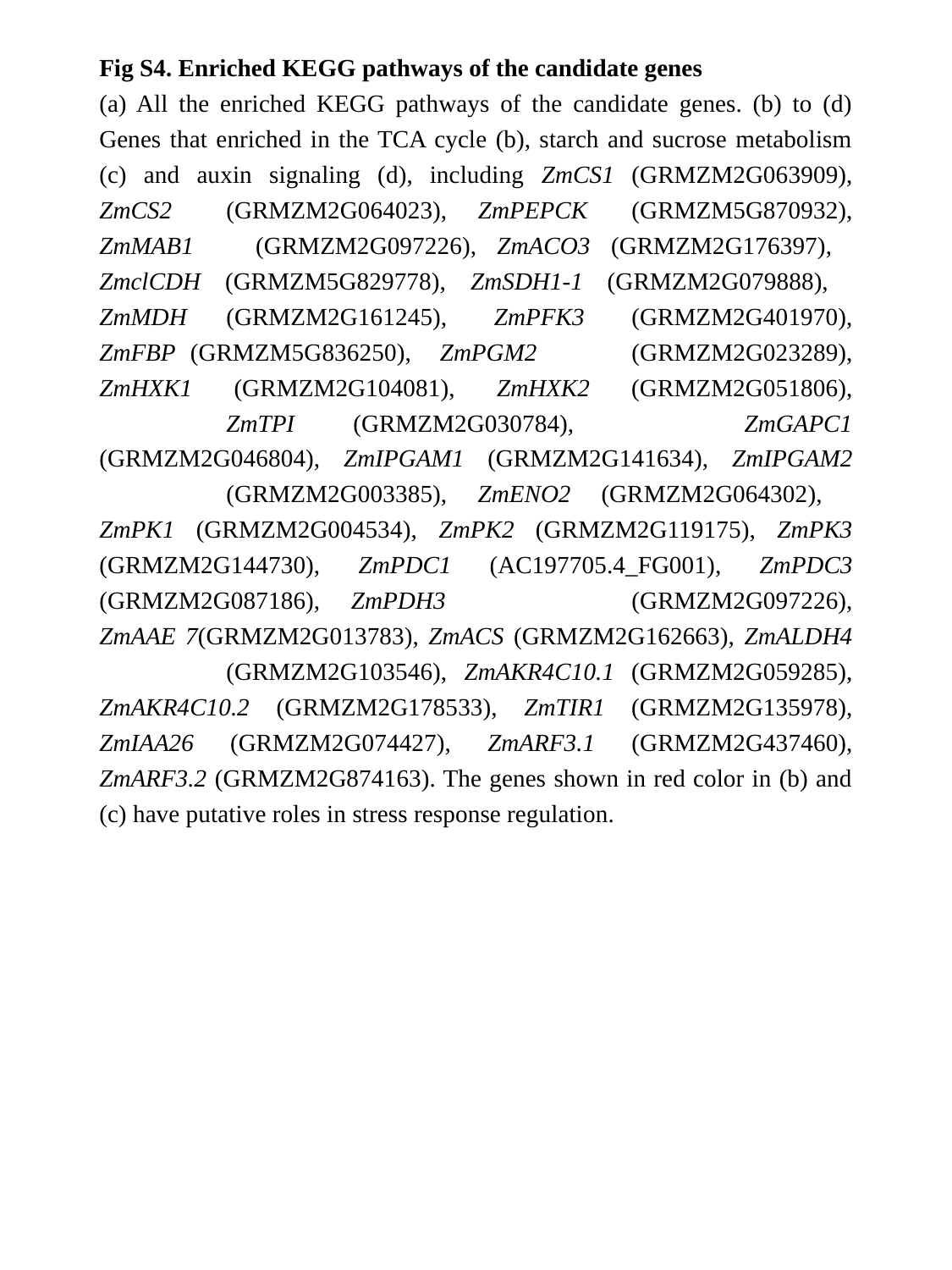

Fig S4. Enriched KEGG pathways of the candidate genes
(a) All the enriched KEGG pathways of the candidate genes. (b) to (d) Genes that enriched in the TCA cycle (b), starch and sucrose metabolism (c) and auxin signaling (d), including ZmCS1 (GRMZM2G063909), ZmCS2	(GRMZM2G064023), ZmPEPCK	(GRMZM5G870932), ZmMAB1 (GRMZM2G097226), ZmACO3 (GRMZM2G176397), ZmclCDH (GRMZM5G829778), ZmSDH1-1 (GRMZM2G079888), ZmMDH	(GRMZM2G161245), ZmPFK3 (GRMZM2G401970), ZmFBP (GRMZM5G836250), ZmPGM2	(GRMZM2G023289), ZmHXK1 (GRMZM2G104081), ZmHXK2 (GRMZM2G051806),	ZmTPI	(GRMZM2G030784), ZmGAPC1 (GRMZM2G046804), ZmIPGAM1 (GRMZM2G141634), ZmIPGAM2 	(GRMZM2G003385), ZmENO2 (GRMZM2G064302), ZmPK1 (GRMZM2G004534), ZmPK2 (GRMZM2G119175), ZmPK3 (GRMZM2G144730), ZmPDC1 (AC197705.4_FG001), ZmPDC3 (GRMZM2G087186), ZmPDH3		(GRMZM2G097226), ZmAAE 7(GRMZM2G013783), ZmACS (GRMZM2G162663), ZmALDH4	(GRMZM2G103546), ZmAKR4C10.1 (GRMZM2G059285), ZmAKR4C10.2 (GRMZM2G178533), ZmTIR1 (GRMZM2G135978), ZmIAA26 (GRMZM2G074427), ZmARF3.1 (GRMZM2G437460), ZmARF3.2 (GRMZM2G874163). The genes shown in red color in (b) and (c) have putative roles in stress response regulation.

## Slide 7
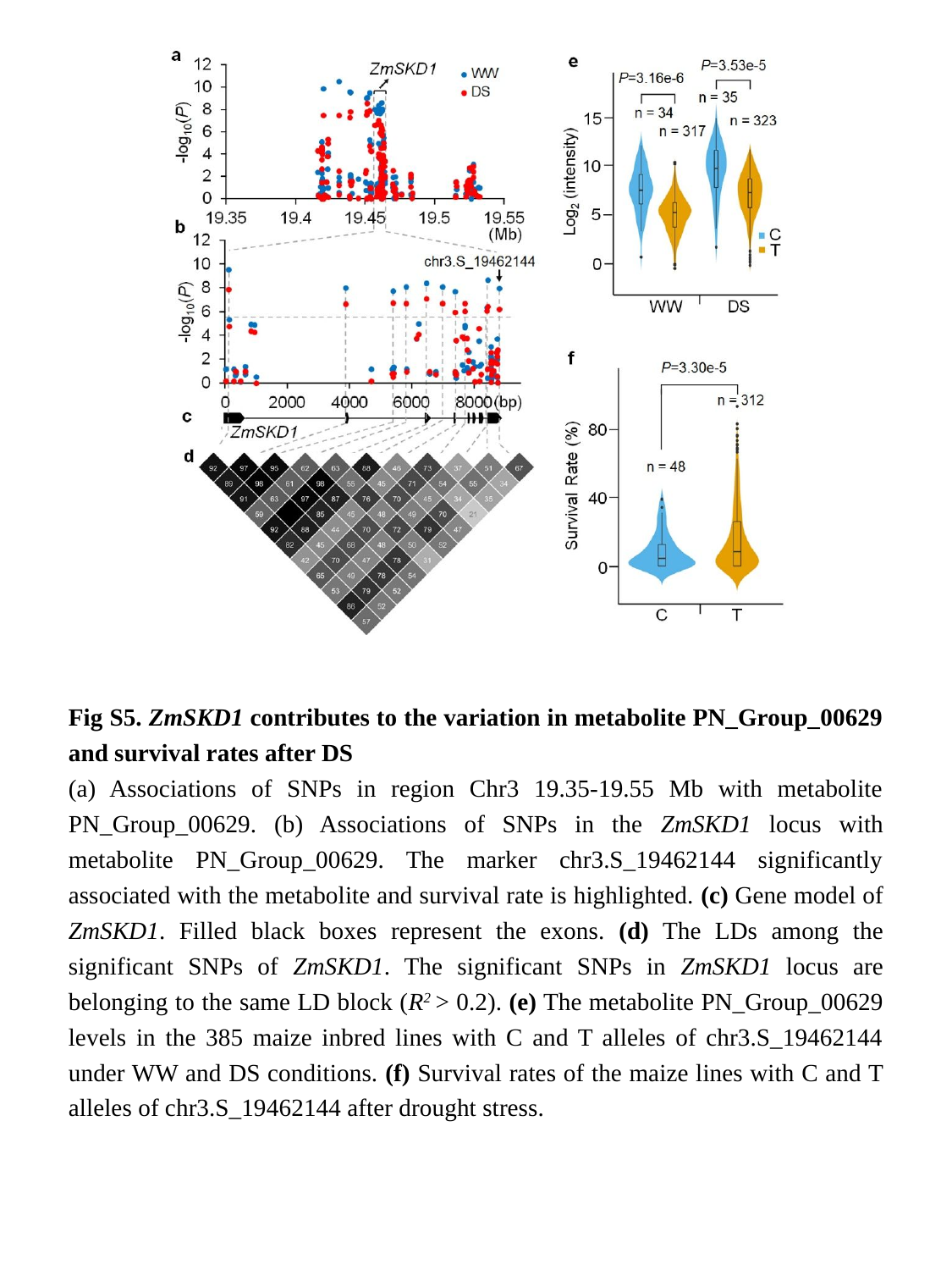

Fig S5. ZmSKD1 contributes to the variation in metabolite PN_Group_00629 and survival rates after DS
(a) Associations of SNPs in region Chr3 19.35-19.55 Mb with metabolite PN_Group_00629. (b) Associations of SNPs in the ZmSKD1 locus with metabolite PN_Group_00629. The marker chr3.S_19462144 significantly associated with the metabolite and survival rate is highlighted. (c) Gene model of ZmSKD1. Filled black boxes represent the exons. (d) The LDs among the significant SNPs of ZmSKD1. The significant SNPs in ZmSKD1 locus are belonging to the same LD block (R2 > 0.2). (e) The metabolite PN_Group_00629 levels in the 385 maize inbred lines with C and T alleles of chr3.S_19462144 under WW and DS conditions. (f) Survival rates of the maize lines with C and T alleles of chr3.S_19462144 after drought stress.

## Slide 8
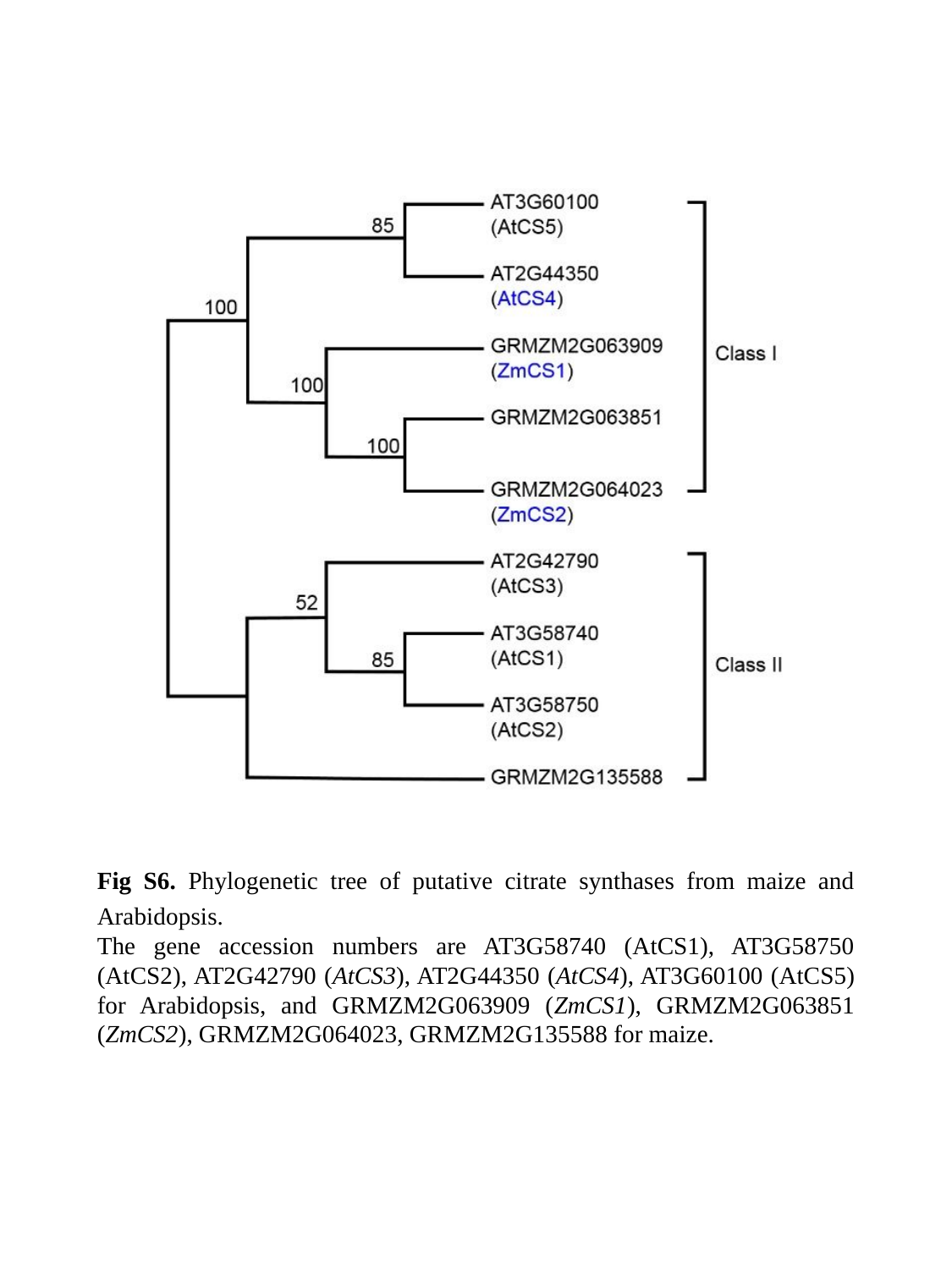

Fig S6. Phylogenetic tree of putative citrate synthases from maize and Arabidopsis.
The gene accession numbers are AT3G58740 (AtCS1), AT3G58750 (AtCS2), AT2G42790 (AtCS3), AT2G44350 (AtCS4), AT3G60100 (AtCS5) for Arabidopsis, and GRMZM2G063909 (ZmCS1), GRMZM2G063851 (ZmCS2), GRMZM2G064023, GRMZM2G135588 for maize.

## Slide 9
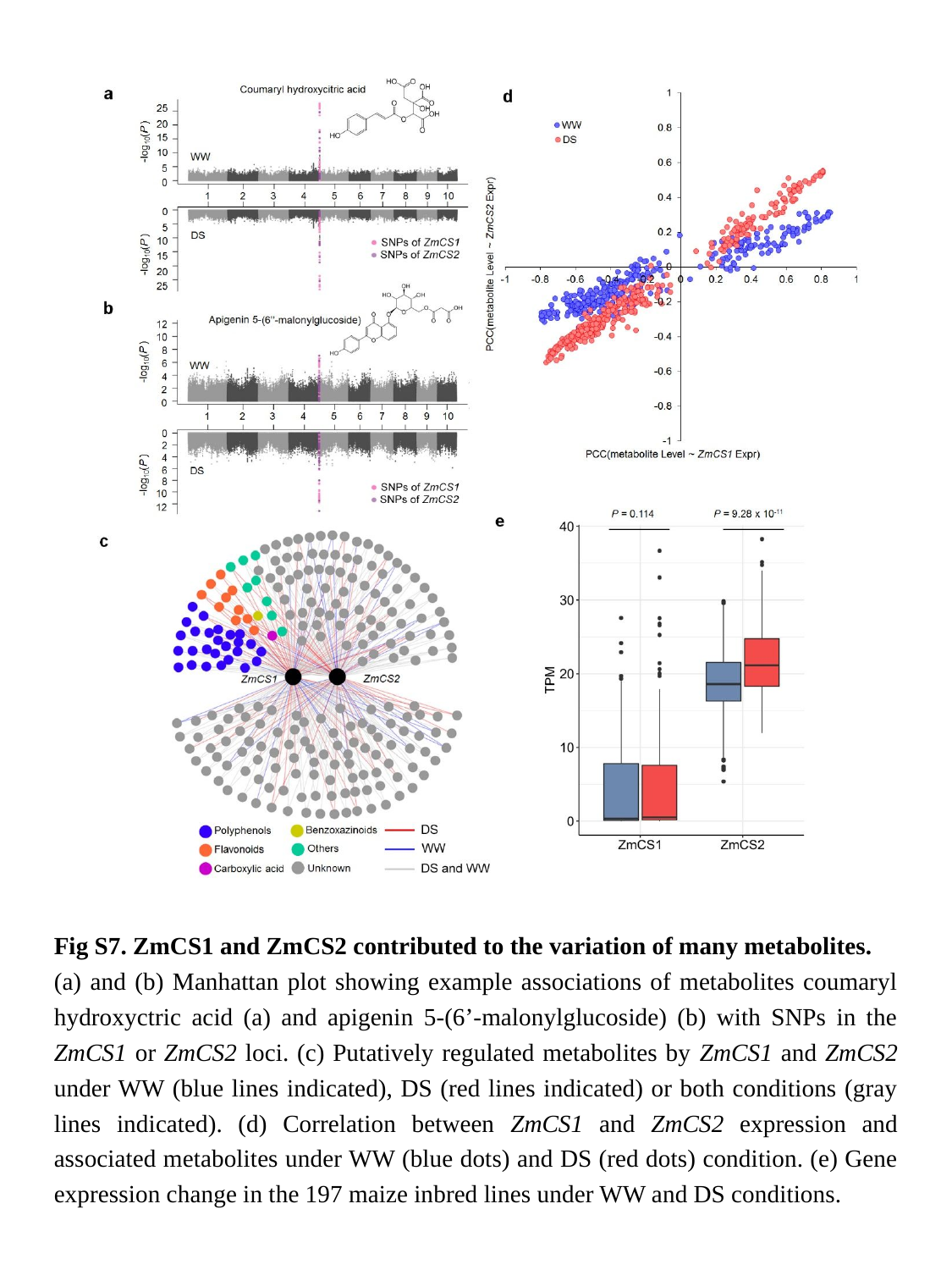

Fig S7. ZmCS1 and ZmCS2 contributed to the variation of many metabolites.
(a) and (b) Manhattan plot showing example associations of metabolites coumaryl hydroxyctric acid (a) and apigenin 5-(6’-malonylglucoside) (b) with SNPs in the ZmCS1 or ZmCS2 loci. (c) Putatively regulated metabolites by ZmCS1 and ZmCS2 under WW (blue lines indicated), DS (red lines indicated) or both conditions (gray lines indicated). (d) Correlation between ZmCS1 and ZmCS2 expression and associated metabolites under WW (blue dots) and DS (red dots) condition. (e) Gene expression change in the 197 maize inbred lines under WW and DS conditions.

## Slide 10
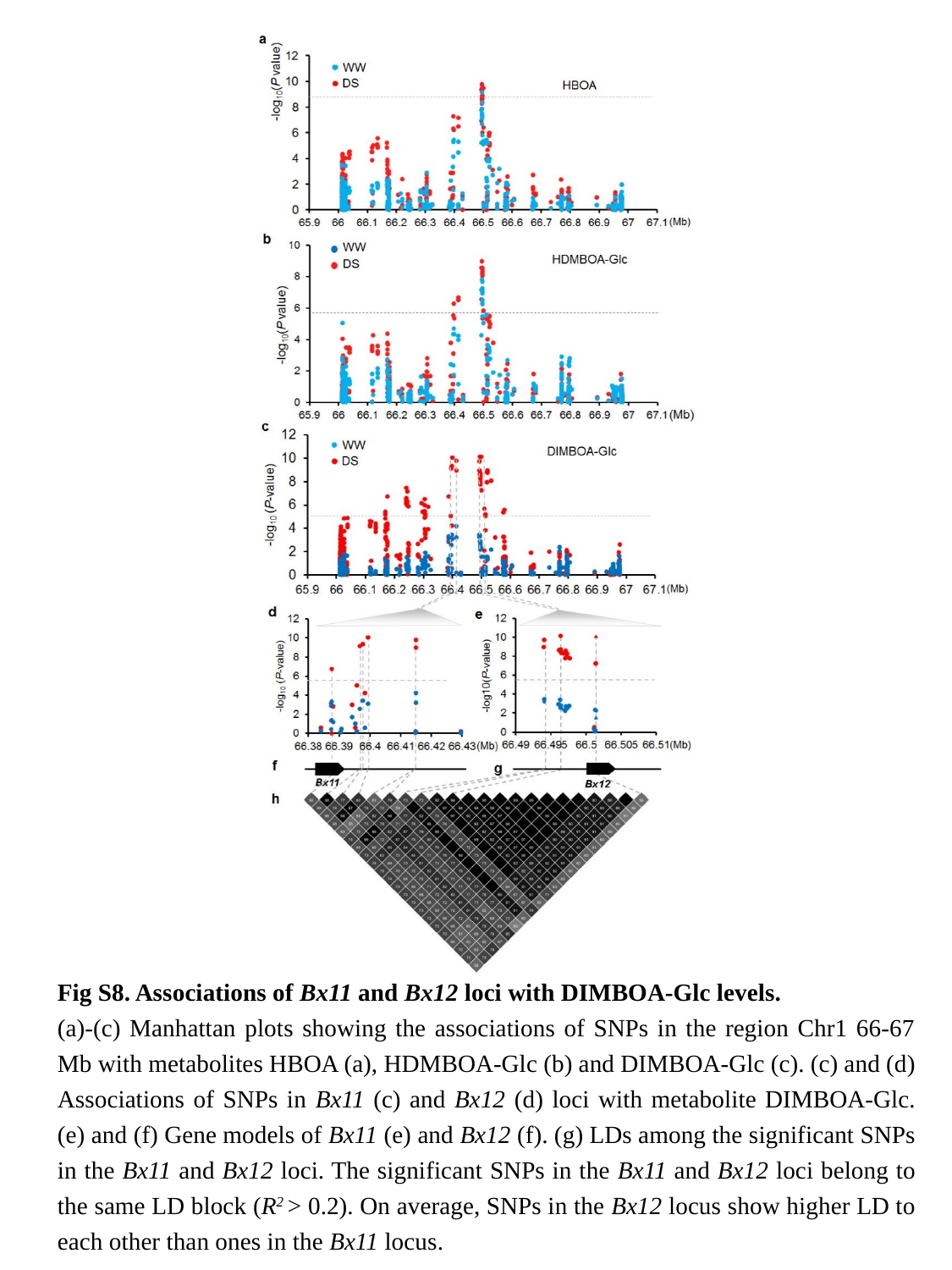

Fig S8. Associations of Bx11 and Bx12 loci with DIMBOA-Glc levels.
(a)-(c) Manhattan plots showing the associations of SNPs in the region Chr1 66-67 Mb with metabolites HBOA (a), HDMBOA-Glc (b) and DIMBOA-Glc (c). (c) and (d) Associations of SNPs in Bx11 (c) and Bx12 (d) loci with metabolite DIMBOA-Glc. (e) and (f) Gene models of Bx11 (e) and Bx12 (f). (g) LDs among the significant SNPs in the Bx11 and Bx12 loci. The significant SNPs in the Bx11 and Bx12 loci belong to the same LD block (R2 > 0.2). On average, SNPs in the Bx12 locus show higher LD to each other than ones in the Bx11 locus.

## Slide 11
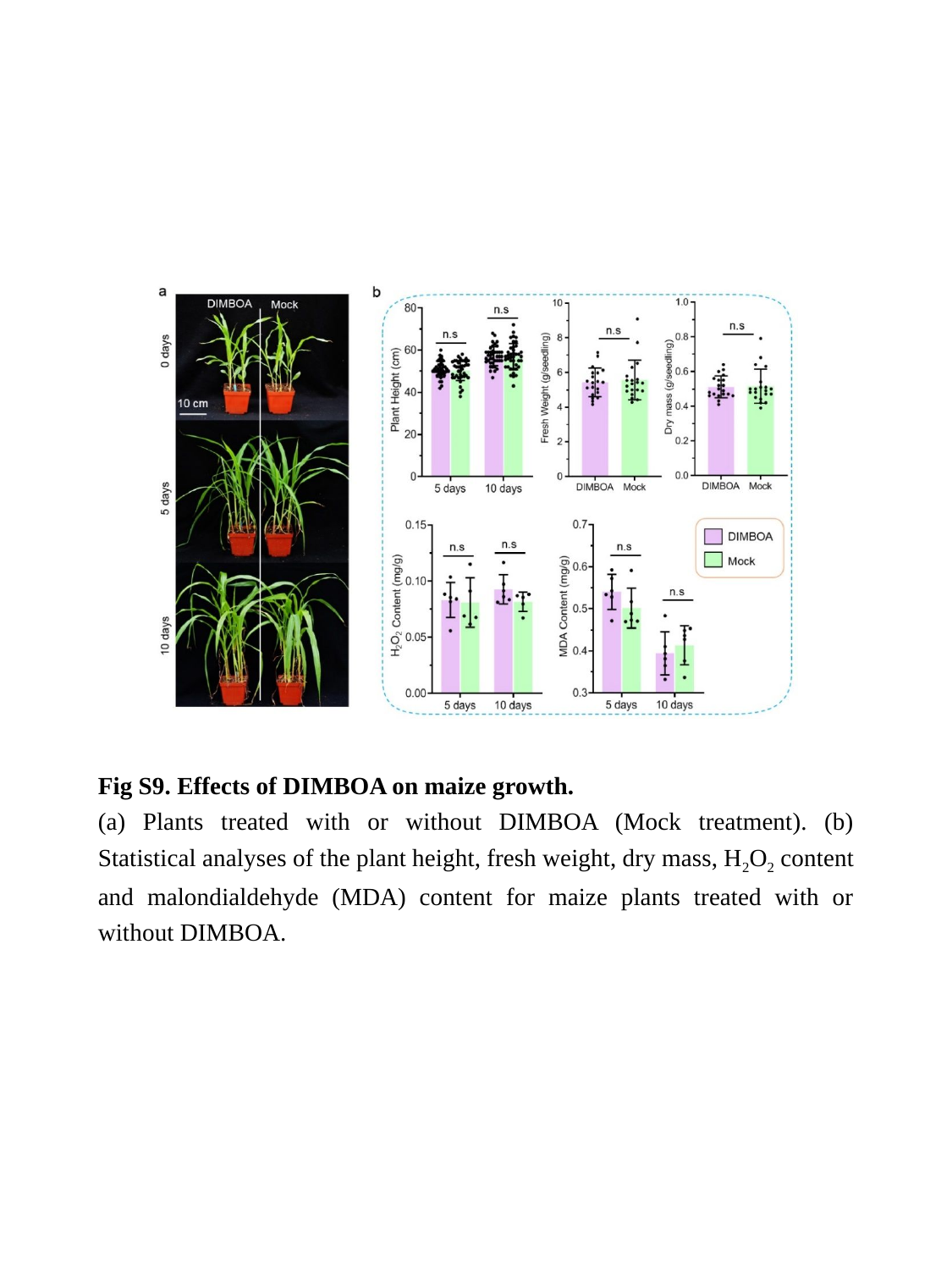

Fig S9. Effects of DIMBOA on maize growth.
(a) Plants treated with or without DIMBOA (Mock treatment). (b) Statistical analyses of the plant height, fresh weight, dry mass, H2O2 content and malondialdehyde (MDA) content for maize plants treated with or without DIMBOA.

## Slide 12
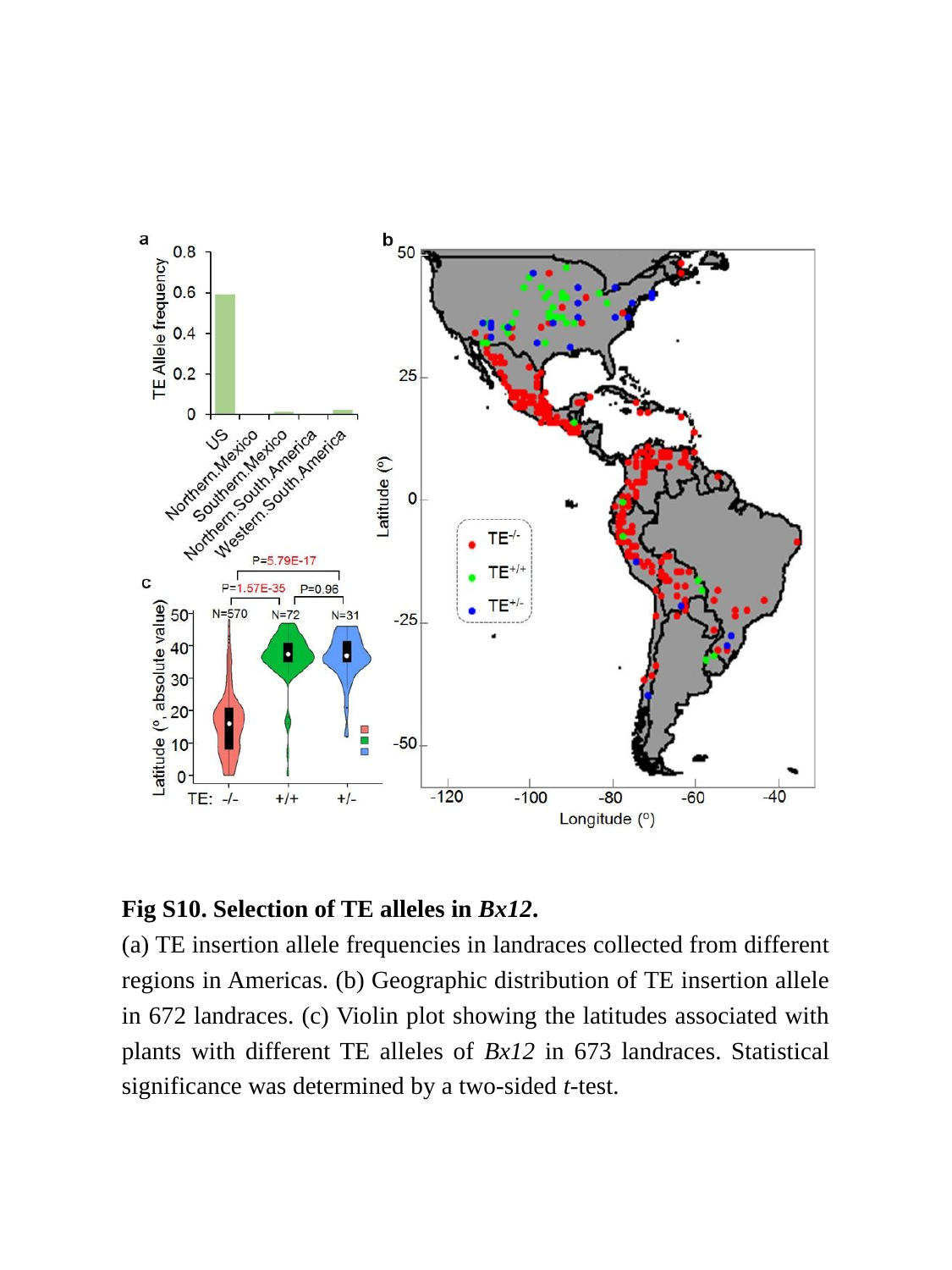

Fig S10. Selection of TE alleles in Bx12.
(a) TE insertion allele frequencies in landraces collected from different regions in Americas. (b) Geographic distribution of TE insertion allele in 672 landraces. (c) Violin plot showing the latitudes associated with plants with different TE alleles of Bx12 in 673 landraces. Statistical significance was determined by a two-sided t-test.

## Slide 13
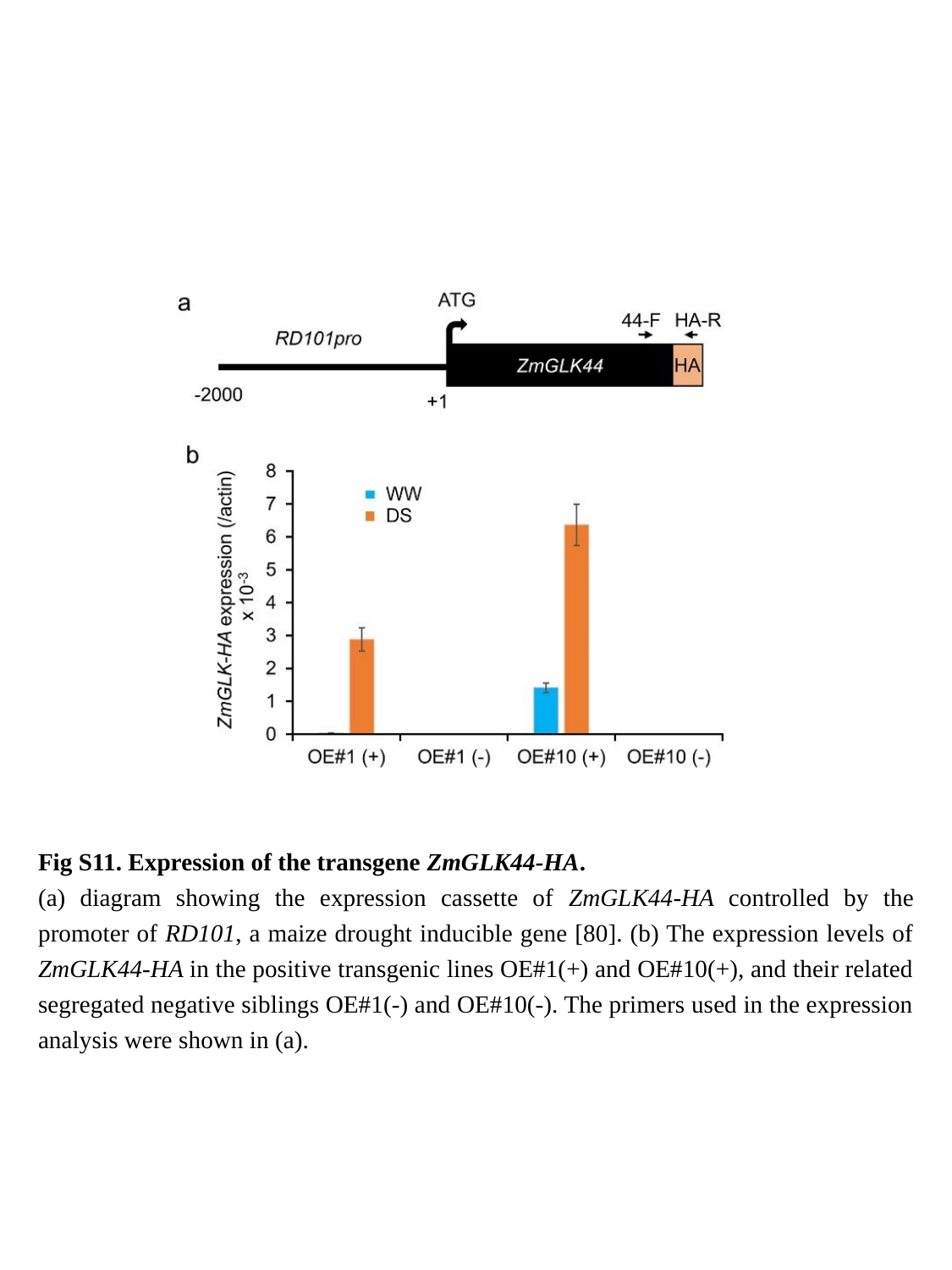

Fig S11. Expression of the transgene ZmGLK44-HA.
(a) diagram showing the expression cassette of ZmGLK44-HA controlled by the promoter of RD101, a maize drought inducible gene [80]. (b) The expression levels of ZmGLK44-HA in the positive transgenic lines OE#1(+) and OE#10(+), and their related segregated negative siblings OE#1(-) and OE#10(-). The primers used in the expression analysis were shown in (a).

## Slide 14
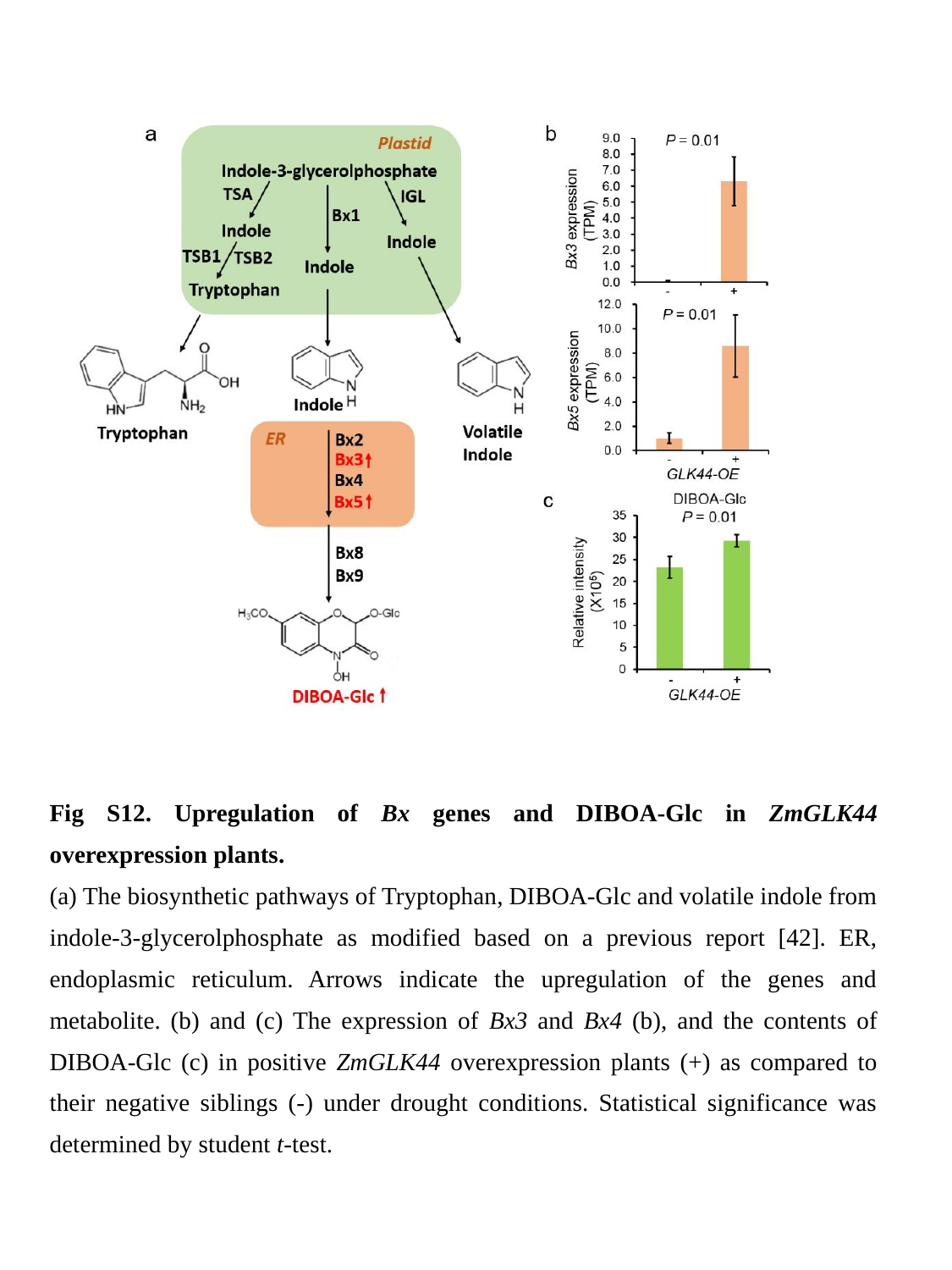

Fig S12. Upregulation of Bx genes and DIBOA-Glc in ZmGLK44 overexpression plants.
(a) The biosynthetic pathways of Tryptophan, DIBOA-Glc and volatile indole from indole-3-glycerolphosphate as modified based on a previous report [42]. ER, endoplasmic reticulum. Arrows indicate the upregulation of the genes and metabolite. (b) and (c) The expression of Bx3 and Bx4 (b), and the contents of DIBOA-Glc (c) in positive ZmGLK44 overexpression plants (+) as compared to their negative siblings (-) under drought conditions. Statistical significance was determined by student t-test.

## Slide 15
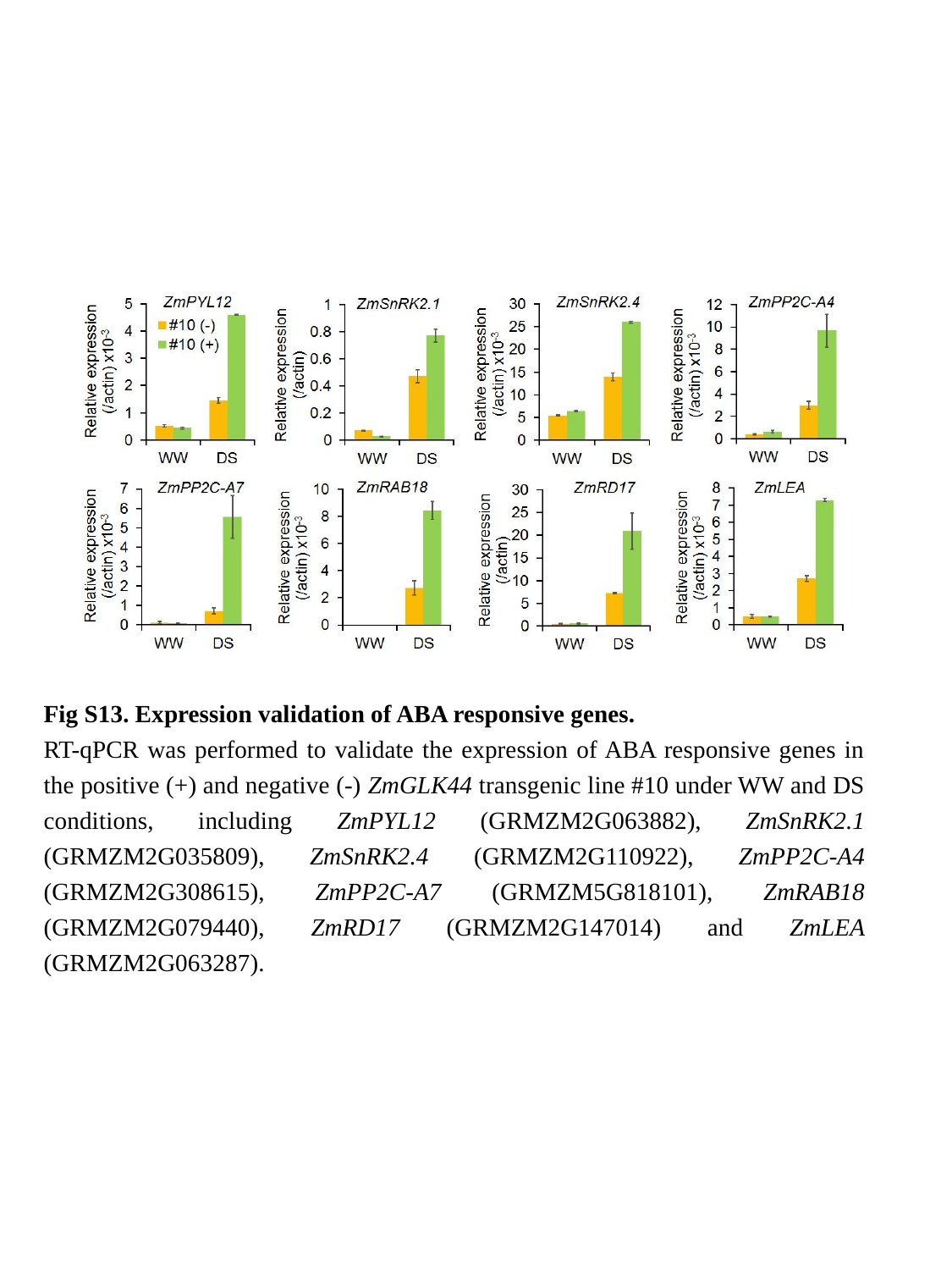

Fig S13. Expression validation of ABA responsive genes.
RT-qPCR was performed to validate the expression of ABA responsive genes in the positive (+) and negative (-) ZmGLK44 transgenic line #10 under WW and DS conditions, including ZmPYL12 (GRMZM2G063882), ZmSnRK2.1 (GRMZM2G035809), ZmSnRK2.4 (GRMZM2G110922), ZmPP2C-A4 (GRMZM2G308615), ZmPP2C-A7 (GRMZM5G818101), ZmRAB18 (GRMZM2G079440), ZmRD17 (GRMZM2G147014) and ZmLEA (GRMZM2G063287).

## Slide 16
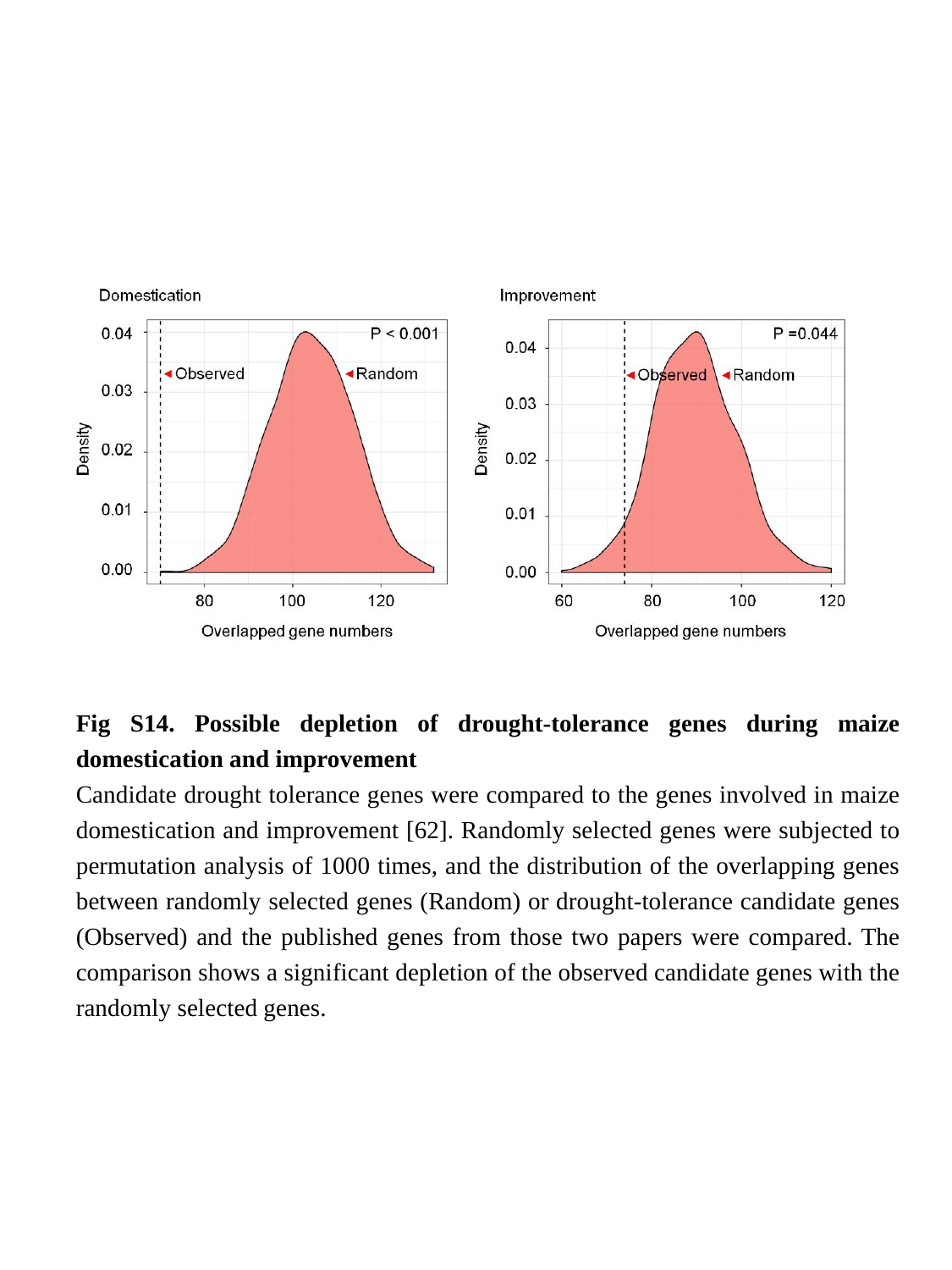

Fig S14. Possible depletion of drought-tolerance genes during maize domestication and improvement
Candidate drought tolerance genes were compared to the genes involved in maize domestication and improvement [62]. Randomly selected genes were subjected to permutation analysis of 1000 times, and the distribution of the overlapping genes between randomly selected genes (Random) or drought-tolerance candidate genes (Observed) and the published genes from those two papers were compared. The comparison shows a significant depletion of the observed candidate genes with the randomly selected genes.
